# Evaluation of two microcosm systems for co-treatment of LDPE_oxo_ and lignocellulosic biomass for biochar production

**DOI:** 10.1186/s40824-021-00222-w

**Published:** 2021-07-02

**Authors:** Alejandra Castillo-Toro, Juan F. Mateus-Maldonado, Diana N. Céspedes-Bernal, Leonardo Peña-Carranza, Adriana I. Páez-Morales, Raúl A. Poutou-Piñales, Juan C. Salcedo-Reyes, Lucía A. Díaz-Ariza, Laura C. Castillo-Carvajal, Aura M. Pedroza-Rodríguez, Luis D. Gómez-Méndez

**Affiliations:** 1 Laboratorio de Microbiología Ambiental y de Suelos, Unidad de Investigaciones Agropecuarias (UNIDIA). Departamento de Microbiología. Facultad de Ciencias. Pontificia Universidad Javeriana, Bogotá, Colombia; 2 Laboratorio de Biotecnología Molecular, Grupo de Biotecnología Ambiental e Industrial (GBAI). Departamento de Microbiología. Facultad de Ciencias. Pontificia Universidad Javeriana, Bogotá, D.C. Colombia; 3 Laboratorio de Películas Delgadas y Nanofotónica. Departamento de Física. Facultad de Ciencias. Pontificia Universidad Javeriana, Bogotá, D.C. Colombia; 4 Laboratorio de Interacciones Planta Suelo Microorganismos (LAMIC), Grupo de Investigación en Agricultura Biológica. Departamento de Biología. Facultad de Ciencias, Bogotá, D.C. Colombia; 5 Facultad de Ciencias de la Salud, Universidad Anáhuac Campus Norte, Huixquilucan, Estado de México Mexico

**Keywords:** Malachite green, Germination test, Plastic roughness, Oxo-biodegradable low-density polyethylene

## Abstract

**Background:**

The co-transformation of solid waste of natural and anthropogenic origin can be carried out through solid-state-fermentation systems to obtain bio-products with higher added value and lower environmental impact.

**Methods:**

To evaluate the effect of *Pleurotus ostreatus* on co-transformation of oxo-degradable low-density polyethylene (LDPE_oxo_) sheets and lignocellulosic biomass (LCB), were assembled two 0.75 L microcosm systems in vertical (VMS) and horizontal (HMS) position. The pre-treated sheets with luminescent O_2_ plasma discharges were mixed with pine bark, hydrolyzed brewer’s yeast and paper napkin fragments and incubated for 135 days at 20 ± 1.0 °C in the presence of the fungus. With the co-transformation residues, biochar (BC) was produced at 300 ± 1.0 °C (BC300) for 1 h, then used to carry out adsorption studies, using the malachite green dye (MG) at pH 4.0, 7.0 and 9.0 ± 0.2. Finally, the biochar was the substrate for the germination of carnation seeds (*Dianthus caryophyllus*) and Ray-grass (*Lolium* sp.) in vitro.

**Results:**

For HMS, the decrease in static contact angle (SCA) was 63.63% (*p* = 0.00824) and for VMS 74.45% (*p* = 0.00219), concerning the pristine. Plastic roughness in VMS was higher (26%) concerning the control. Throughout the 135 days, there were fungal growth and consequently laccase (Lac), manganese peroxidase (MnP) and lignin peroxidase (LiP) activities. During the first 75 days, CO_2_ production increased to 4.78 ± 0.01 and 4.98 ± 0.01 mg g-1 for HMS and VMS, respectively. In MG adsorption studies, the highest amount of the colourant adsorbed at both pH 4.0 and 7.0 ± 0.2.

**Conclusions:**

Finally, the biochar or the biochar enriched with low concentrations of plant growth-promoting microorganisms and inorganic fertilizer favours the germination of *Dianthus caryophyllus* and *Lolium* sp., seeds.

**Supplementary Information:**

The online version contains supplementary material available at 10.1186/s40824-021-00222-w.

## Introduction

Polyethylene (PE) is a plastic insulating material for electrical conductivity, malleable and hydrophobic [1]. Have been estimated that from 1950 to 2015, about 8.3 billion metric tons of plastic products, including PE, were produced, of which 6.3 billion metric tons became solid waste, of which 12% incinerated, while the remaining 79% was disposed of in landfills or inappropriately ended up in water bodies and natural ecosystems [2].

According to PE density, it can be classified as high-, low- and linear low-density. Low-density polyethylene (LDPE) is composed of short chains of amorphous aliphatic carbon, joined by C-C bonds [1, 3]. LDPE is crucial in the manufacture of single-use products, such as bags, cigarettes, packaging, cutlery, etc. The excessive use and production of these non-degradable products have led to their accumulation; consequently, their control, proper disposal and waste treatment become very difficult. Besides the illegal dumping of solid waste, landfills also influence the accumulation of LDPE in natural ecosystems due to torrential rains and landslides, among others [4]. The harmful effect of oxo-degradable low-density polyethylene (LDPE_oxo_) on ecosystems result from improper disposal of plastic waste after use. For example, the emergence of the SARS-CoV-2 virus, which causes Covid-19, in December 2019, increased the use of personal protective accessories (PPA), especially gloves and face masks, and, in the same vein, the generation of more plastic waste [5, 6], some of which are construct from LDPE or LDPE_oxo_.

Trying to solve this problem pro-oxidant additives are incorporated during LDPE manufacture, generating the LDPE_oxo_. This plastic maintains the characteristics of no additives LDPE; however, pro-oxidant additives help accelerate its deterioration [7, 8] by modifying the thermal and mechanical properties of the traditional plastic to increase the susceptibility to photo- and thermo- oxidation of the polymer [9]. However, the incorporation of pro-oxidants does not guarantee the complete transformation or mineralization; it only favours the fragmentation of the LDPE [7, 10–15].

Therefore, the development of technologies to contain, control and accelerate the degradation of LDPE_oxo_ are address to mitigate the environmental impact. Some research focused on generating ecological alternatives to promote the LDPE_oxo_ degradation has shown that the pretreatment of plastic by using complementary physical, chemical or biological processes favours the transformation [12–14, 16]. The pretreatment of LDPE_oxo_ with oxygen plasma discharges is a viable alternative [12], since it generates some polymeric materials structural modifications [17–19] and increases the hydrophilicity; favouring colonization, growth and material biotransformation when utilizing microorganisms [12, 14].

Biotransformation of LDPE with *Pleurotus ostreatus* has previously reported by different authors [12, 14, 16]. They demonstrated that *P. ostreatus* has several mechanisms to biotransform the LDPE. The first one is related to abundant filamentous growth, which allows the colonization of *P. ostreatus* on different organic and inorganic solid materials. Additionally, it produces small double-walled conidia of independent propagation units to mycelium [20–23].

On the other hand, the fungus produces a wide range of enzymes related to the biodegradation of lignin (ligninases E.C. 1.11.1.14), cellulose (cellulases E.C. 3.2.1.4) and hemicellulose (hemicellulases E.C. 3.2.1.). These enzymes under natural conditions are responsible for the hydrolysis of polymers and also acting in the biotransformation of LDPE and bioplastics containing starch or cellulose in their structure [24]. During the hydrolytic activity mediated by cellulases and hemicellulases, H_2_O_2_ is produced, which participates in the activation of peroxidase (E.C 1.11.1.7) and takes part in biological Fenton processes. Through Fenton processes, are formed hydroxyl radicals with high oxidizing power, low selectivity and complement the oxidation of natural polymers and LDPE [12, 14, 16, 23, 25].

The development of technologies to favour the colonization, growth and enzyme production by *P. ostreatus* for the biotransformation of LDPE and LDPE_oxo_, is a challenge for biotechnology. It involves interdisciplinary work to guarantee that the mycelium stays in contact with the plastic and use it as a carbon, nitrogen and trace elements source in sufficient concentration to support the primary metabolism. Besides physical and chemical factors such as temperature, pH, humidity, particle size, and aeration must be kept under control [22, 26, 27].

In this sense, different methodologies and assays scales have reported, ranging from experiments in Petri dishes [12], microcosm systems [14], a combination of aerobic/anaerobic processes [28], composting [29], among others.

Solid-state fermentation (SSF) for the production of *P. ostreatus* as an edible fungus is a technology that can be modified for use in the co-transformation of organic solid waste, specifically associated with lignocellulosic biomass [30] and LDPE/LDPE_oxo_ [14]. Within lignocellulosic biomasses, sawdust by-products such as sawdust, bark and shavings, alone or mixed with agro-industrial by-products, are very useful substrates for the production of *P. ostreatus* biomass and could be used as economical filling materials or mixture for the co-transformation of plastic. It is an eco-friendly technology because, in a single process, two types of waste (with different complexity and chemical structure) are bio-transformed to obtain bioproducts with high added value, such as fungal biomass, enzymes, and raw materials for biofuels production [14, 30–33].

To applying dual-purpose technology, there are two options, the SSF in vertical or horizontal systems. In vertical systems, the reactors are closed column-shaped, which allow the control of the operating conditions during the process. However, system problems are the scaled-up and the biotransformation efficiency [34]. The horizontal systems (also called tray-type bioreactors) are frequently for the production of edible fungi. In this system, the substrate is distributed horizontally and generates a big surface contact area for oxygen transfer, heat and elimination of gases produced by microbial metabolism [35]. The use of one or the other systems depends on the purpose since the conditions that favour the co-transformation of the two types of carbon-rich waste but with different chemical structure must be controlled.

In the present work, the co-transformation of O_2_-plasma-pretreated LDPE_oxo_ and LCB using *P. ostreatus* was evaluated in two microcosm systems. The aim was to promote the biodegradation of LDPE_oxo_ using as co-substrate lignocellulosic biomass (LCB) formed by pine bark, hydrolyzed beer and paper napkins. Additionally, within the framework of solid waste management and its use, biochar (BC) was produced and characterized from the by-products of SSF to be evaluated as an adsorbent of Malachite Green (MG) dye and as a substrate for the “in vitro” germination of carnation (*Dianthus caryophyllus*) and Ray-grass (*Lolium* sp.).

## Materials and methods

### Pretreatment to LDPE_oxo_ sheets

LDPE_oxo_ sheets (3.0 ± 0.1 cm long and 1.0 ± 0.1 cm wide) were assayed for hydrophobicity, roughness, presence of functional chemical groups by static contact angle (SCA), and atomic force microscopy (AFM) techniques, following the methodology previously proposed by Gómez-Méndez et al. [12]. Once characterized, they were subjected to low-temperature plasma discharges (LTP) with O_2_ in a cylindrical jar-type chamber of 18 cm high by 18 cm in diameter with disc electrodes separated by 5.6 cm and coupled to a turbo-molecular vacuum pump (Pfeiffer Vacuum). After generating a vacuum in the chamber, plasma treatment started, using the conditions previously reported by Gómez-Méndez et al. [12].

### Microorganism and inoculum production

*Pleurotus ostreatus* strain (CMPUJH124) was reactivated and propagated on wheat bran agar supplemented with chloramphenicol (wheat bran (Toning) 175 gL^− 1^, glucose 10 gL^− 1^, yeast extract 2 gL^− 1^, peptone 5 gL^− 1^, MgSO_4_ 7H_2_O 0.05 gL^− 1^, MnSO_4_ H_2_O 0.076 gL^− 1^, KH_2_PO_4_ 0.1 gL^− 1^, chloramphenicol 0.1 gL^− 1^, agar-agar 20 gL^− 1^) following conditions reported by Moreno-Bayona et al. [14]. The inoculum production as a fungal biomass pellet was in the same liquid culture medium for 10 days, 30 °C at 120 rpm. The biomass recovery was by centrifugation at 9790 x *g*, 10 min at 4.0 ± 1.0 °C, using a Sorvall™ RC 6 plus centrifuge and was washed five times under sterile conditions to eliminate residues from the culture medium; the first four times with 0.85% (w/v) saline and the last one with a 0.625 gL^− 1^ glucose solution and 0.050 gL^− 1^ NH_4_Cl [14].

### Co-transformation of LDPE_oxo_ sheets and LCB in microcosm systems

**Fig. 1:**
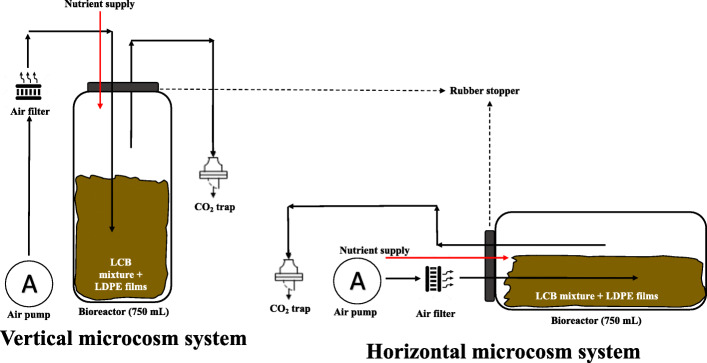
Diagram of the VMS and HMS used in this study

For co-transformation of LDPE_oxo_ sheets (pre-treated with O_2_ plasma) and LCB with *P. ostreatus*, were assembled two 0.75 L microcosm systems. The first placed vertically (vertical microcosm system - VMS) and the other horizontally (horizontal microcosm system - HMS). In each of the containers, a mixture of LCB composed of 24.0 ± 0.1 g of pine bark, 27.0 ± 0.1 g of chopped paper napkins and 9.0 ± 0.1 g of hydrolyzed brewer’s yeast was added, weighed on a scale with an error percentage of 0.001%, brand Ohaus™. Each of the microcosms with the LCB was sterilized three times during 60 min, at 121 °C and 0.5 atm of pressure [14]. Under sterile conditions, four pre-treated LDPE_oxo_ sheets, 3 mL of trace nutrient solution (0.625 gL^− 1^ glucose, KH_2_PO_4_ 2 gL^− 1^, NH_4_Cl 0.05 gL^− 1^, MgSO_4_ 0.5 gL^− 1^, CaCl_2_ 0.1 gL^− 1^, CuSO_4_ 1.5 gL^− 1^, 2, 2-Azino-bis (3-ethylbenzothiazoline-6-sulfonic acid) (ABTS) 0.05 gL^− 1^) and 3 g of wet pelleted *P. ostreatus* biomass were added [14]. Subsequently, after material homogenization, each experimental unit was sealed with a rubber stopper with three ports for air injection, nutrient supply and CO_2_ capture, using a trap with NaOH 0.4 N solution. The aeration supplied was done by a Xilong® AP-005 motor (110 V-60 Hz) with an intermittent flow of 135 L min^− 1^. Aeration was performed three times a week for 1 h. Finally, every 15 days, 3 mL of a trace nutrient solution (glucose 0.625 gL^− 1^, KH_2_PO_4_ 2 gL^− 1^, NH_4_Cl 0.05 gL^− 1^, MgSO_4_ 0.5 gL^− 1^, CaCl_2_ 0.1 gL^− 1^, CuSO_4_ 1.5 gL^− 1^, ABTS 0.05 gL^− 1^) was added [14, 31], (Fig. 1). Were assembled fifteen VMS microcosms and 15 HMS microcosms; the evaluation was 135 days long. Samples were taken in triplicate by sacrifice (3 VMS microcosms and 3 HMS microcosms removed at each sampling times, 0, 35, 75, 107 and 135 days). As an absolute control, the same sterile filling mixture was used but not inoculated with *P. ostreatus*.

In each sampling, were extracted the entire LCB colonised with *P. ostreatus* (residual lignocellulosic biomass - R-LCB) and the LDPEoxo sheets to analyse the response variables. LDPEoxo followed by hydrophobicity, roughness, AFM [14]. For R-LCB, moisture percentage, pH [36], total organic carbon (TOC), [37] and CO_2_ production [31, 38]. Also, laccase (Lac, EC. 1.10.3.2), [39], manganese peroxidase (MnP, EC. 1.11.1.13), [40] and lignin peroxidase (LiP, EC. 1.11.1.14) activities were quantified [41].

For these determinations, a solid-liquid extraction was performed with the addition of 50 mM sodium acetate buffer (pH 5.0 ± 0.2, 0.735% (w/v) of citric acid monohydrate C_6_H_8_O_7_ H_2_0, 1.912 (w/v) of trisodium citrate dihydrate C_6_H_9_Na_3_O_7_.2H_2_O) and Tween 80 at 0.01% (v/v) in a 1:5 ratio. The extraction was maintained in agitation at 200 rpm for 5 h; subsequently, filtered on Whatman No. 3 paper and centrifuged at 8000 x *g* for 15 min to obtain a raw extract at which assayed enzymatic activities [14, 31]. The degree of condensation of R-LCB, associated with the formation of fulvic (FS) and humic (HS) substances, was also monitored [14, 42, 43], (Supplementary Material S1).

### Production and characterization of biochar using the R-LCB

To take advantage of the R-LCB resulting from VMS and HMS a pyrolysis process was performed at 300 ± 1.0 °C for 1 h to obtain biochar. It was produced by placing lots of 62.75 ± 0.01 g of R-LCB, without LDPE_oxo_ sheets, in aluminium containers with a capacity of 100 g and drying at 70 °C for 12 h in an electric oven HACEB™. The container was then placed in a bell at Anaerogen™ for 12 h at 19 ± 1.0 °C to lower the oxygen concentration [14]. For pyrolysis, it was placing the aluminium trays with the R-LCB inside a muffle Labtech™ for 1 h at 300 ± 1.0 °C. Once the process finished, the biochar was preserved in Anaerogen™ bells to make the same characterizations as R-LCB, also, of fixed carbon (FC, %), volatile carbon (VC, %), ashes (%), [37] and biochar performance [44].

### Adsorption studies

The adsorption studies performed by using biochar and MG (triphenylmethane dye). Measurement of adsorption kinetics was at pH 4.0, 7.0 and 9.0 ± 0.2, preparing 50 mL vials Schott™ in triplicate containing 20 mL of MG dye solution with 15 mg L^− 1^ of initial concentration. Later, after the addition of 0.5 g of biochar, maintained in constant agitation at 120 rpm and 19 ± °C. Samples were taken at the beginning 5, 10, 15, 20, 30, 40, 50, 60, 90, 120, 150 and 250, minutes to determine the absorbance at 618 nm and convert it to MG concentration (mg L^− 1^). These values allow calculation of the *q* value (amount of dye adsorbed in mg g^− 1^ of biochar (Eq. 1).

1 $$ q=\frac{V\left({C}_0-{C}_f\right)}{X} $$

**Where**: *V* is the volume of the solution (in L), C_f_ is the final concentration of the MG (mg L^− 1^), *C*_*o*_ is the initial concentration of the MG (in mg L^− 1^), X is the grams of biochar.

The *q* value used in each case corresponds to the average *q* value of 3 replicates of the same pH value (4.0, 7.0 and 9.0 ± 0.2). Subsequently, pseudo-first-order, pseudo-second-order, and Elovich models allow to evaluating which one best describes the adsorption kinetics of MG to biochar. The calculated parameters were the maximum amount of dye in mg adsorbed per gram of biochar (*qe*), pseudo-first-order adsorption coefficient (*k*), pseudo-second-order adsorption coefficient (*k*^*2*^), initial adsorption coefficient (*α*) and desorption coefficient (*β*), [45, 46].

### In vitro seed germination studies using biochar

Finally, biochar served as the substrate for the germination “in vitro” of carnation seeds (*Dianthus caryophyllus*) and Ray-grass (*Lolium* sp.). The assay included four treatments and one control: co-inoculated biochar with Plant Growth-promoting bacteria (PGPB) at a concentration of 1.0 × 10^7^ Colony Forming Unit per millilitre (CFU mL^− 1^) (T1), co-inoculated biochar with PGPB and chemical fertilizer Nutriponic™ (T2), biochar with only Nutriponic™ (T3), biochar with only sterile distilled water (T4). The control contained sterile-peat alone, hydrated with distilled water. For PGPB, inoculum production followed the methodology reported by Rojas-Higuera et al. [31]. The culture was incubated for 12 h at 30 ± 2.0 °C, with constant agitation of 120 rpm, using a thermostatted horizontal agitator New Brunswick™ Innova 44. The inoculum-concentration was accessed using decimal dilutions and seeding at the surface of 0.1 mL in nutritive agar. The boxes were incubated for 12 h at 30 ± 2.0 °C, using an incubator Memmert™.

For each experimental-units assembly were used 60 × 15 mm Petri dishes containing 2.0 ± 0.5 g of 2 mL hydrated biochar (at field capacity) and PGPB inoculum (1.0 × 10^7^ CFU mL^− 1^) or sterile distilled water (T4). Regarding the chemical fertilizer Nutriponic™, 10 μL of the product were applied to the 2 g of biochar to have the commercial concentration recommended by the producer (100 mg L^− 1^). Then, were placed nine seeds from each of the plants to be evaluated in each Petri dish. The Petri dishes were hatched in the dark for 5 days at 19 ± 2.0 °C. At the end of the period, the germination index (GI %) was calculated by following the previously reported methodology [45, 46].

### Statistical analysis

The T-student test allowed for analysing the biotransformation success of the LDPE_oxo_ sheets. The analysis of variance (ANOVA) followed by post-hoc multiple comparisons (Tukey’s test) enabled studying the enzyme production, the respirometry of the system, and the lignocellulosic biomass characteristics. Besides, was performed a test for homogeneity of variances (Levenne’s test). For seed germination, the comparison of means between treatments used the SAS 9.0 software.

## Results

### Biotransformation of LDPE_oxo_ sheets in VMS and HMS

**Fig. 2:**
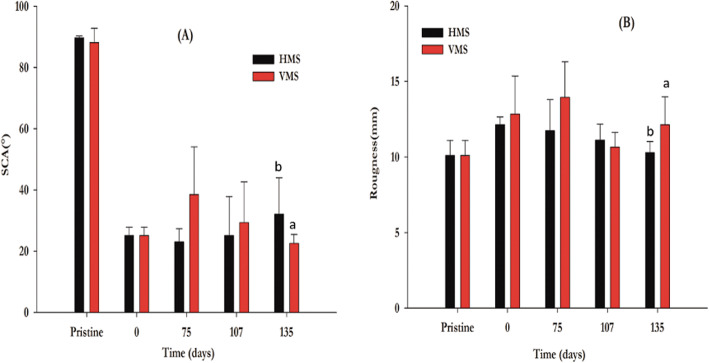
Measurement of SCA (**A**) and roughness (**B**), in VMS and HMS. The letter above bars in Figs. (A) and (B) indicate the presence of heterogeneous groups, hence significant differences

During 135 days of treatment with *P. ostreatus*, the hydrophobicity of the LDPE_oxo_ sheets was determined in HMS and VMS employing SCA. Before being treated with O_2_ plasma, the LDPE_oxo_ sheets (pristine) presented an SCA of 88.3 ± 2.9 °. At the end of the treatment, changes in hydrophobicity were observed, both in HMS, (32.11 ± 8.38) °, and in VMS (22.56 ± 2.89) °. For HMS, the decrease in SCA was 63.63% (*p* = 0.00824) and for VMS was 74.45% (*p* = 0.00219), concerning the pristine (Fig. 2A).

**Fig. 3:**
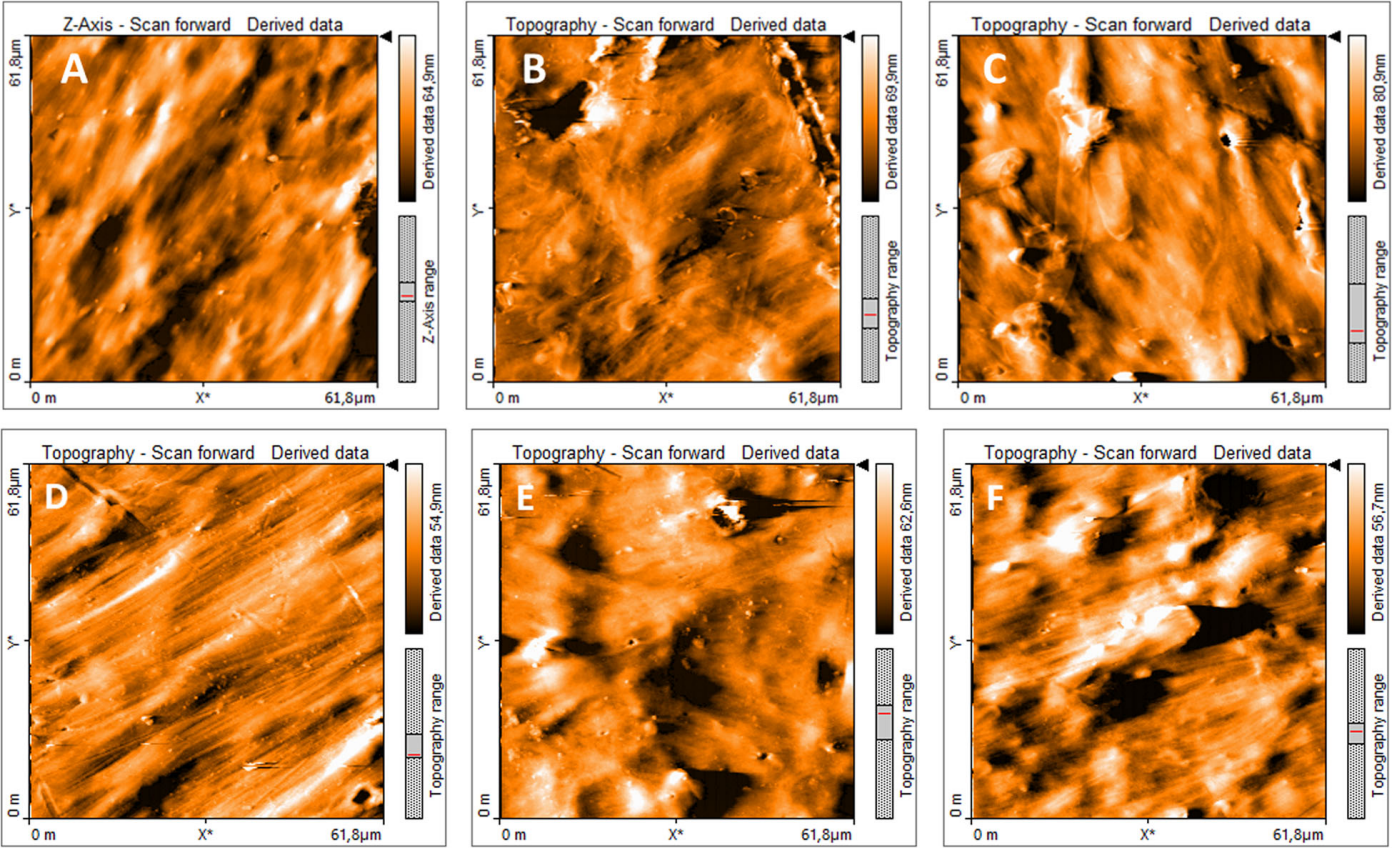
AFM of LDPE_oxo_ sheets. **A** Pristine LDPE_oxo_ from VMS. **B** LDPE from VMS (day 107), where cracks and holes caused by the plasma synergistic effect are observed - *P. ostreatus.* **C** LDPE_oxo_ from VMS (day 107), where hyphae of *P. ostreatus* and micropores are perceived. **D** Pristine LDPE_oxo_, from HMS. **E** LDPE from HMS (day 107), where a littles holes caused by the plasma synergistic effect are observed - *P. ostreatus*. **F** LDPE_oxo_ from HMS (day 107), where hyphae of *P. ostreatus* and micropores are perceived

The initial roughness of the pristine sheets of both microcosm systems was (10.1 ± 1) nm. After 135 days of treatment with *P. ostreatus* in HMS, the roughness of the LDPE_oxo_ sheets was 10.29 ± 0.73 nm, (*p* = 0.3428); the percentage change was only 1.75% (Figs. 3E, F). In contrast, the final roughness of the sheets in the VMS was 13.62 ± 0.931 nm, (*p* = 0.02534), with a 26% change, concerning the pristine (Fig. 2B). Additionally, when analyzing the AFM images, cracks and voids caused by the plasma synergistic effect - *P. ostreatus* were observed at 107 days of evaluation (Figs. 3B, C).

### Biotransformation of LCB into HMS and VMS

**Fig. 4:**
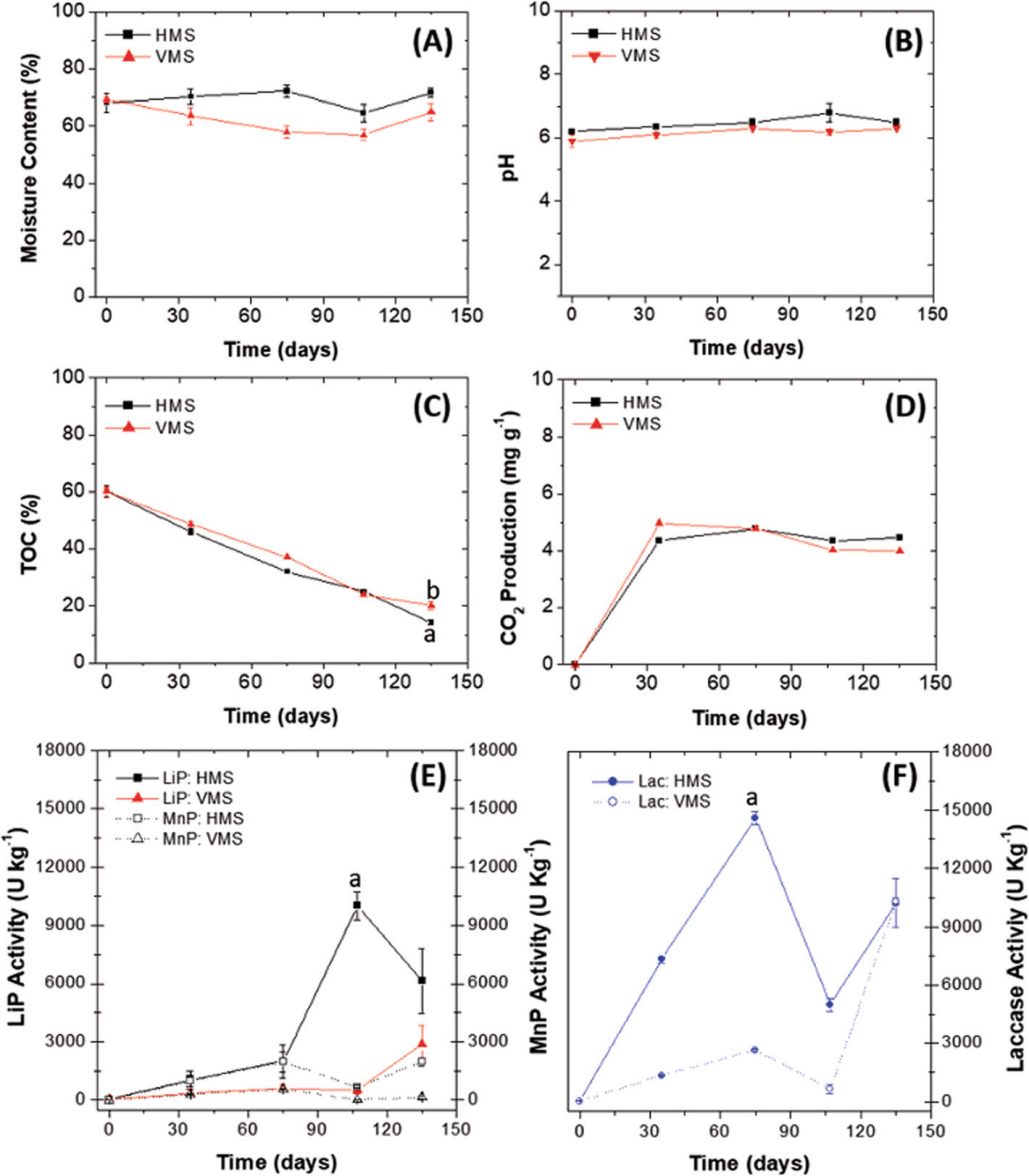
Analysis of the HMS and VMS microcosm for 135 days. Moisture content (%) (**A**), pH (**B**), TOC (%) (**C**), CO_2_ production (mg g^− 1^) (**D**), Lac activity (U Kg^− 1^) (**E**) and LiP and MnP ligninolytic activity (U Kg^− 1^) (**F**). The results presented in the figures correspond to the average of three replicates. Letters represent Tukey homogeneous subsets. a* corresponds to the best treatment

In the same way that in microcosm systems, the biotransformation of the LDPE_oxo_ sheets occurred, LCB also experienced chemical and physical changes associated with the biological activity of *P. ostreatus*. The initial humidity was similar for both microcosm systems obtaining values of 68.1 ± 3.3 and 69.3 ± 1.1%, for HMS and VMS, respectively. In the HMS no losses in the humidity percentage were observed, on the contrary, it increased to finish in 71.7 ± 1.5% after 135 days. VMS showed a slight decrease in the percentage of humidity that oscillated between 63.5 ± 6.5 and 65 ± 1.9% at 35 and 135 days, respectively (Fig. 4A). The pH was 6.2 ± 0.1 and 5.9 ± 0.2 for HMS and VMS, respectively. During the 135 days, no significant differences were observed for the pH (*p* > 0.0001), so that during the whole experiment, the acidity conditions favoured the growth of the fungal mycelium and the enzymatic activity (Fig. 4B).

The percentage of TOC and CO_2_ production are indicators of biotransformation and depend on the biological activity of *P. ostreatus* [47]. The initial TOC values were similar for the two types of microcosms (60.2 ± 2.1 and 60.7 ± 1.7) for HMS and VMS, respectively. In both systems, a progressive decrease of this variable occurred, and significant differences were only found between systems (*p* = 0.043) at 135 days with values of 14.2 ± 0.2 and 20.1 ± 1.3 for HMS and VMS, respectively, being more efficient the transformation of carbon into HMS (Fig. 4C). In HMS, the TOC decrease was higher because the LCB layer was thin, facilitating the colonization of fungal mycelium, the air, heat and mass transfer. Consequently, the fermentation process was more efficient [35], while in VMS, the microcosm creates a thick layer of LCB that decreases the contact surface available for colonization, oxygen gradients can form that hinder and delay the growth of *P. ostreatus* [22, 48].

The production of CO_2_ is a variable that allows us to identify whether biodegradation processes associated with the metabolic activity of *P. ostreatus* are taking place in both the LCB and the LDPE_oxo_ sheets. During the first 75 days, the CO_2_ production increased to 4.78 ± 0.01 and 4.98 ± 0.01 mg g^− 1^ for HMS and SMV, respectively. Later, it decreased to 4.47 ± 0.01 and 4.02 ± 0.01 mg g^− 1^ for HMS and VMS, at 135 days, respectively (Fig. 4D).

The expectation regarding the initial (day 0) enzyme activity in both microcosm systems was equal to zero; however, the enzymatic activity was: for LiP 63.75 ± 27.6 and 79.68 ± 55.208 U Kg^− 1^, for HMS and SMV, respectively; for MnP 25.4 ± 9.78 and 23.701 ± 0.543 U Kg^− 1^, for HMS and SMV, respectively and Lac 41.1 ± 16.57 and 41.145 ± 4.475 U Kg^− 1^, for HMS and SMV, respectively; this result could be because part of the medium, wheat bran and enzymes produced during propagation were absorbed by the mycelium of pelletized *P. ostreatus*, which also promoted the enzymatic activity quantified on day 0.

LiP and MnP showed significantly higher activity, in HMS, during the 135 days of evaluation (*p* = 0.0026 and *p* = 0.031 for LiP and MnP respectively), obtaining the highest values at 107 days for LiP with 10,008 ± 706 U Kg^− 1^ and at 135 days for MnP with 1962.4 ± 220.3 U Kg^− 1^. In the VMS, the highest LiP and MnP activities were 2868 ± 941 U Kg^− 1^ (day 135) and 576 ± 30 U Kg^− 1^ (day 75) (Fig. 4E). Similar behaviour was observed with Lac in the HMS, being higher than in the VMS (*p* < 0.0007) with 14,599 ± 320 U Kg^− 1^, while the activity in the VMS did not exceed 10,314 ± 190 U Kg^− 1^ (Fig. 4F).

In both the HMS and VMS systems, the measurement of ultraviolet-visible spectra (UV-VIS) spectra made it possible to study the humification degree of the LCB, which is qualitatively related to the generation of FS and humic HS substances (Supplementary Material S2). Besides the absorption in the visible region of visible spectra (V), were calculated the condensation/polymerization ratios (E_4_/E_6_). These allow a semi-quantitative evaluation of the degree of humification of humic substances extracted from organic materials with different degrees of transformation. In abiotic control, the E_4_/E_6_ ratio ranged from 1.09 ± 0.23 to 2.67 ± 0.92, for HMS and VMS, respectively. These were the lowest of all calculated E_4_/E_6_ ratios (Table 1). In the initial HMS and VMS systems and inoculated with *P. ostreatus*, the E_4_/E_6_ ratios calculated for FS were 2.74 ± 0.97 and 2.59 ± 1.09, respectively. The values obtained for HS were similar for the two systems evaluated, with values of 2.60 ± 0.15 and 2.20 ± 0.33, respectively. After 135 days of biotransformation, the E_4_/E_6_ relations for FS and HS increased notably concerning to the initial ones, with values of 8.58 ± 0.97, 13.84 ± 1.16, 3.07 ± 0.35 and 3.66 ± 0.92 for FS and HS in HMS and VMS, respectively. These findings proved that in both systems, the indirect humification or condensation of the FS and HS initiated and that it still requires time to reach the complete maturation of the biotransformed LCB, which would imply the decrease of the HS ratios below 2.0 (Table 1).

**Table 1:** E_4_/E_6_ Ratio of fulvic acids (FA) and humic acids (HA) isolated from Horizontal Microcosm System (HMS) and Vertical Microcosm System (VM)

	HMS		VMS	
	E_**4**_/E_**6**_		E_**4**_/E_**6**_	
**Time (d)**	**FS**	**HS**	**FS**	**HS**
Abiotic control	2.33 ± 0.51	2.67 ± 0.92	2.33 ± 0.11	2.67 ± 0.54
0	2.74 ± 0.97	2.60 ± 0.15	2.59 ± 1.09	2.20 ± 0.33
135	8.58 ± 0.97	3.07 ± 0.35	13.84 ± 1.16	3.66 ± 0.92

*E*_*4*_*/E*_*6*_ Abs_465nm_/Abs_665nm,_ *HMS* Horizontal Microcosm System, *VMS* Vertical Microcosm System

### Production and characterization of biochar

In general, the raw material (RM) shows a pH of 5.70 ± 0.14, humidity of 6.90 ± 0.35%, and TOC of 45.1 ± 5.2%. Further analysis showed that RM has a low percentage of FC (1.2 ± 0.4) %, with a high content of VC (85.1 ± 2.0) %, and ash concentration was 13.7 ± 2.0%.

Once produced the biochar, a decrease in the humidity, TOC, and VC percentages occurred, obtaining values about 4.7 ± 0.16%, 29.5 ± 1.3%, and 73.2 ± 9.0%, respectively. On the contrary, the pH, FC and ash content increased with values of 6.1 ± 0.1, 6.55 ± 0.12% and 20.2 ± 1.9%, respectively. According to the carbon content (29.5 ± 1.3%), the biochar was classified in class III (Total Carbon (TC) > 10 and < 30%).

### Adsorption studies

**Fig. 5:**
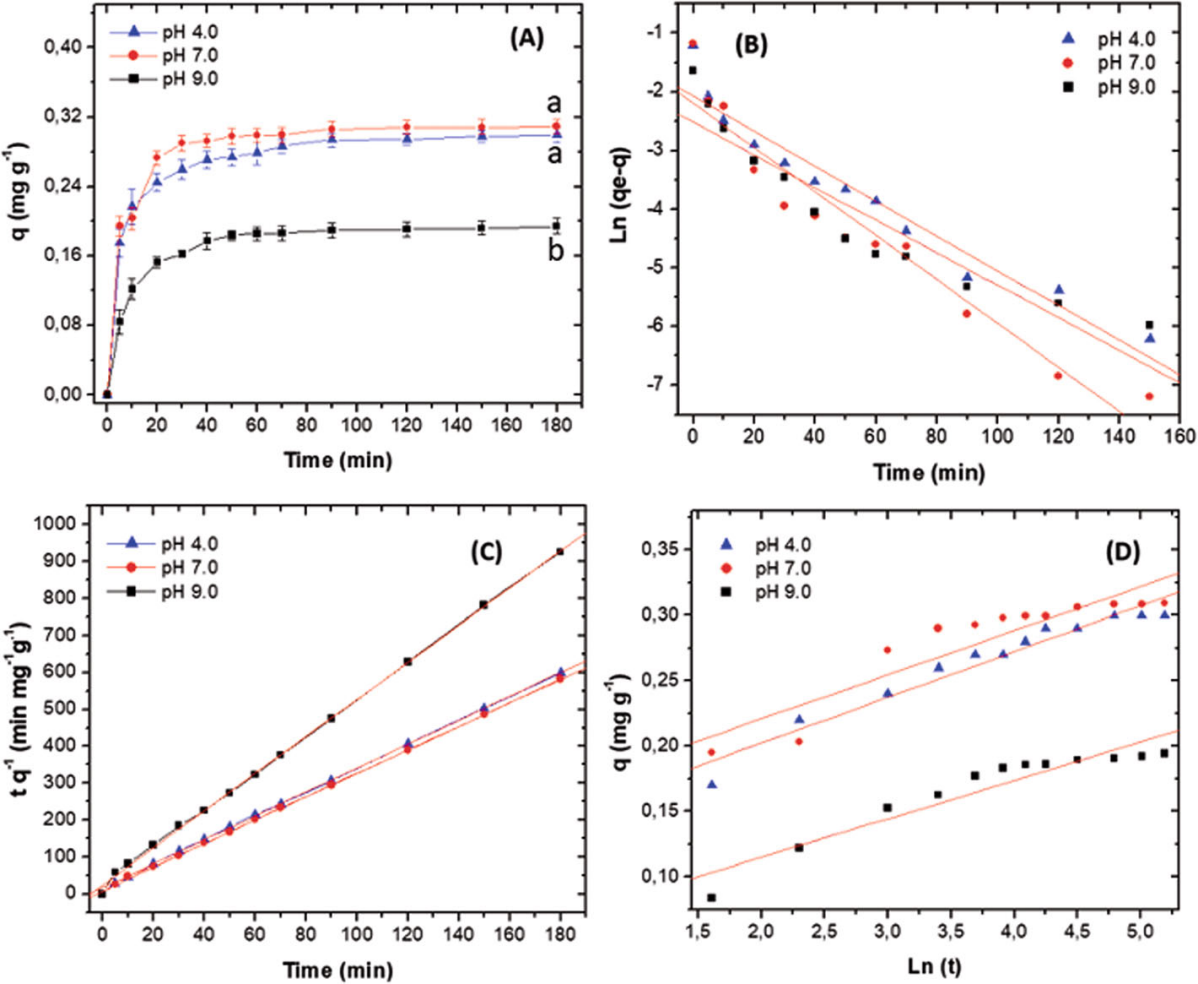
**A** *q* value versus time at different pH*.* **B** Model of pseudo-first-order. **C** Model of pseudo-second-order. **D** Model of Elovich in the function of time for Malachite Green. Results presented correspond to the mean of three replicas. (note: regression date is available in (Table 3)

The largest amount of MG adsorbed were at pH 4.0 and 7.0 ± 0.2, reaching adsorption/desorption equilibrium between 30 and 60 min of contact, being significantly different than at pH 9.0 ± 0.2 (*p* < 0.0001). Between pH 4.0 and 7.0 ± 0.2 no significant differences were observed (*p* > 0.0001). At pH 9.0 ± 0.2, less dye adsorption occurred and reached the balance after 80 min. The values of *qe* expressed as mg g^− 1^ were 0.321 ± 0.003, 0.311 ± 0.023 and 0.194 ± 0.045 for pH 4.0, 7.0 and 9.0 ± 0.2, respectively (Fig. 5A).

The values of *qe* obtained at the three pH were applied to the pseudo-first-order, pseudo-second-order and Elovich models. Figure 5B, C, D and Table 2 present the results for each of the models. For the pseudo-first-order model, the values of *R*^2^ were 0.9491, 0.9376 and 0.8977 for pH 4.0, 7.0 and 9.0 ± 0.2. The values of *qe* calculated by the model were similar to the experimental *qe* values, and the adsorption constants for pseudo-second-order appears in Table 2. The constants were higher at pH 4.0 and 7.0 ± 0.2, suggesting that, at low pH, the number of active sites available for dye adsorption increases. For the pseudo-second-order model, the values of *R*^2^ were higher than 0.9990 for the three pH, and the adsorption constants of pseudo-second-order were higher at pH 4.0 and 7.0 ± 0.2 (0.241 and 0.354 g mg^− 1^ min^− 1^ respectively). At pH 9.0 ± 0.2 the value of the constant was 0.204 g mg^− 1^ min^− 1^ (Fig. 5C, Table 2). When treating the experimental data with Elovich’s model, *R*^2^ was lower (0.8398, 0.8593 and 0.9316, for pH 4.0, 7.0 and 9.0 ± 0.2, respectively). Concerning α and β, the highest values were at pH 4.0 and 7.0 ± 0.2 (Fig. 5C, Table 2). For this model, the lowest coefficients were at pH 9.0 ± 0.2.

**Table 2:** Rate Constants of pseudo-first-order, pseudo-second-order and Elovich models for BC300 at pH of 4.0, 7.0 and 9.0 ± 0.2

pH ± 0.2	Pseudo-first-order			Pseudo-second-order			Elovich		
	*qe* mg g^−1^	*k* g mg ^− 1^ min^− 1^	*R*^2^	*qe* mg g^− 1^	*k*^*2*^ g mg ^− 1^ min^− 1^	*R*^2^	α mg g^−1^ min^− 1^	β g mg ^− 1^	*R*^2^
4.0	0.323	0.0298	0.9491	0.305	0.242	0.9993	63.457	0.0521	0.8398
7.0	0.316	0.0375	0.9376	0.314	0.354	0.9995	67.949	0.0542	0.8593
9.0	0.197	0.0278	0.8977	0.198	0.204	0.9995	53.940	0.0369	0.9316

Analysing the three models was found that low pH favoured adsorption, and possibly the adsorption of dye by biochar was due to chemisorption. At pH 9.0 ± 0.2, charge repulsion is generated; for this reason, adsorption was lower.

### Seed germination tests

The use of biochar and biochar enriched with PGPB and/or chemical fertilizers as the substrate for seed germination was evaluated for 5 days. With *Dianthus caryophyllus* seeds, were observed significant differences between T1, T2 and T3 treatments concerning the control (*p* < 0.0001). Treatment T4 showed no statistically significant differences (*p* > 0.0001) compared to the experiment control. Among the treatments T1, T2 and T3, T2 and T3 stood out as the best (100%), which contained biochar, PGPB and Nutriponic (T2), while T3 only had the chemical fertilizer Nutriponic (Table 3).

**Table 3:** Seed germination index Test for 5 d at 19 °C

Treatments and control	***Dianthus caryophyllus*** seed	***Lolium*** sp., seed
	Germination index (%)	Germination index (%)
T_1_	98 ± 1.3^b^	**100 ± 5**^**a**^
T_2_	**100 ± 2**^**a**^	**99** ± 4^b^
T_3_	**100 ± 3**^**a**^	**100 ± 8**^**a**^
T_4_	67 ± 4.72^c^	89 ± 2^**c**^
Control	66 ± 2.34	90 ± 5

T_1_: Biochar and PGPB. T_2_: Biochar, PGPB and Nutriponic. T_3_: Biochar and nutriponic. T_4_: Biochar hydrated with water. C: Peat hydrated with water. Letters in the table indicate presence of heterogeneous groups, hence significant differences (*p* < 0.05)

About the trials with *Lolium* sp. seeds, significant differences observed between T1, T2, T2 and the control with peat obtaining 100% germination after 5 days (*p* < 0.0001) (Table 3).

## Discussion

### Biotransformation of the LDPE_oxo_ films in the VMS and HMS

The initial low hydrophobicity (day 0) of the LDPE_oxo_ films in both microcosm systems was due to the treatment with O_2_ plasma [12], which incorporates oxygen, polar and hydrophilic functional groups on the LDPE_oxo_, which favoured the material surface transformation [12, 18, 49]. However, after plasma treatment, the sheets can regain hydrophobicity after 10 days of exposure to O_2_ plasma [49]. In this work, the LDPE_oxo_ sheets post-plasma treatment and after 135 days with *P. ostreatus* in the microcosms did not return to their initial SCA value. Due to a synergy between photodegradation and biodegradation generated by the fungus enzymatic activity. In the VMS, the contact between the microorganism and the plastic sheets was limited, compared to HMS. In HMS, the mixture homogeneity and the contact surface increased as a consequence of fungus-free radial growth that extends to the width. *P. ostreatus* colonised the material, forming a network of hyphae that prevented the material from regaining its initial hydrophobicity. The carbonyl and hydroxyl groups generated on the surface of the material after exposure to O_2_ plasma changed the surface charge of the polymer, favouring the interaction between the cationic functional groups of the fungal wall and the anions on the LDPE_oxo_ surface. Therefore, facilitating the adsorption and subsequent adhesion of *P. ostreatus* to the LDPE_oxo_ sheets [12], also increasing the roughness. Additionally, after adhesion, fungus secretes oligomers and polysaccharides that covalently bind to the material surface, which is relevant since these substances function as carriers of the enzymes produced by *P. ostreatus* to the LDPE_oxo_. Furthermore, the enzymatic attack of polyphenol oxidase (C.E. 1.11.1.7) and peroxidase (C.E. 1.14.18.1) generates free radicals on the surface of LDPE_oxo_, which are vulnerable to oxidative attack [12, 13, 25]. The enzymatic attack and subsequent oxidative attack, break the bonds of the LDPE_oxo_, causing the material to maintain the acquired hydrophilicity after the plasma discharge. Since the biodegradation of the material responds to oxidative reactions, there must be a wide contact surface between the available oxygen and the fungus. Oxygen is involved in the breathing process by *P. ostreatus* and the oxidation of substrates, as this process is a reaction coupled with the reduction of O_2_ [50]. In HMS, the contact surface between oxygen, the microorganism and the substrates were greater; for this reason, the SCA value was lower and maintained for 107 days, compared to the SCA results of VMS at day 135, the SCA in VMS was lower. These results show that both microcosm systems had a synergy between plasma and fungal, thus indicating that the system geometry influences the trend and standard deviation of the response variables associated with the plastic.

Plasma treatment is particularly effective on LDPE_oxo_ sheets, as the pro-oxidant additives favour the formation of pores in the plastic structure; facilitating the penetration of the plasma through the structure; generating hydrophilicity on the material surface [49], and increasing its roughness, as observed in the AFM images (Fig. 3).

### Evaluation of transformation systems in microcosms

Biotransformation of solid organic and inorganic materials (with different complexity degrees and chemical structure) is a strategy for LCB transformation or LCB and plastics co-transformation and also for the production of edible ligninolytic fungi. A strategy that is often carried out by SSF. SSF can be an open, closed, static or rotary manner [14, 21, 26, 31, 51]. In this work, a modified SSF was used to improve the process conditions, favour the fungus metabolism, and increase biotransformation and growth efficiency [12, 20, 52]. In terms of process improvement and control of microcosm systems, it is possible to modify the way LCB is mixed (napkins, brewery by-products, LDPE plastic, etc.) and distributed with other materials within the closed system. To treat the LCB can horizontally be arranged, to perform a thin layer with greater surface area, to accelerates the colonization and growth of the fungi, simulating the Dutch production system for edible fungi or Tray (Tray bioreactor) type reactors [27, 35]. On the other hand, the LCB also can be vertically arranged within a closed system or in plastic bags to form a cylinder of greater height and variable diameter, to simulates the French production system or the packaged bed reactor for SSF [22, 27, 34].

Associated with these modifications in the present work, the effect of horizontal and vertical closed systems on the percentage of humidity in the treatment of LDPE_oxo_ and LCB sheets at laboratory scale in microcosms was evaluated. Concerning the humidity percentage, the initial values were slightly low (68.1 ± 3.3 and 69.3 ± 1.1) %, for HMS and VMS, respectively), (Fig. 2A) if compared with the optimal values reported for a solid fermentation with ligninolytic fungi (70–80%), [26, 27]. However, this did not affect the process of colonization and growth of *P. ostreatus* in any of the microcosm systems; on the contrary, in the HMS, there was an increase in the percentage of humidity (71.7 ± 1.5%) at 135 days, which is related to the highest growth of *P. ostreatus*, since approximately 50% of the humid weight of *P. ostreatus* is water [14, 22]. Additionally, in HMS, the LCB formed a thinner layer than in VMS, generating more surface area, which facilitated oxygen transfer and helped dissipate other gases produced during metabolism [22]. Maintaining humidity between 60 and 80% helps the growth of the fungus and the production of ligninolytic and hydrolytic enzymes (LiP, MnP, Lac, cellulases, hemicellulases, among others); favouring the delignification process of the LCB, under oxidative conditions [22, 26].

The conditions of initial substrate acidity (6.2 ± 0.1 and 5.9 ± 0.2) and final acidity (6.5 ± 0.2 and 6.3 ± 0.5) for HMS and VMS, respectively, were another factor that allowed the abundant development of fungal mycelium and extracellular enzyme production (Fig. 4B). The values remained similar because there was no relationship between the products associated with carbon and nitrogen metabolism. On the one hand, the enzymes endoglucanases (E.C. 3.2.1.4) and endoxylanases (EC 3.2.1.8) were able to release glucose and xylose, which once assimilated by *P. ostreatus* generated organic acids that slightly lowered the pH in the first days of the process. On the other hand, when the mineralization or ammoniation of the organic nitrogen source (hydrolyzed brewer’s yeast) occurred, NH_4_ is produced and increase the pH, without ending up in the alkalinity range (> 7.0). Yoon et al. (2014), reported that in SSF of fungi for production of cellulases must start at pH below 7.0 ± 0.2, and acidification occurs during fermentation. At the end of the process, the pH can return to neutrality and even rise to a pH of 8.0 ± 0.2 [27]. Other authors also reported that culture of *P. ostreatus* in SSF and under similar conditions to those of this work, the pH should be 6.0 ± 0.2, to favour the initial production of mycelium and the later formation of the fruiting body, when the pH is between 3.5 and 5.0 ± 0.2 [48]. The biotransformation of the LCB was demonstrated with the decrease in the percentage of TOC and increase in the production of CO_2_, being higher in the HMS with an initial TOC of 60.2 ± 2.1% and final of 14.2 ± 0.2%. In the VMS, the initial TOC was 60.7 ± 1.7 and the final was 20.1 ± 1.3%, (Fig. 4C and D). In both systems the mixture of residues (pine bark, paper napkins and hydrolyzed brewer’s yeast) allowed the process of colonization and growth of *P. ostreatus* to take place. Being a saprophytic fungus must extract nutrients from the substrate, therefore, the initial colonization and extension of its hyphae are essential for obtaining the source of carbon, nitrogen and trace elements, which is favoured when using mixtures of substrates with different degrees of complexity and not just lignin since, It represents a lower energy expenditure to hydrolyze simpler polymers to obtain carbon as an energy source, than to delignify the phenylpropane subunits strongly bound by C-C and ether bonds, to obtain aliphatic or three-carbon intermediates that can enter the Krebs cycle [14, 53]. On the other hand, one reason why the decrease in TOC was higher in HMS is that, having the LCB distributed in a thin layer *P. ostreatus* colonized the superficial part more quickly and later its hyphae extended towards the middle and lower part of the LCB layer, while in VMS, the LCB formed a thicker cylinder-like layer, decreasing the superficial area for colonization and despite the use of intermittent forced aeration, oxygen gradients could be formed in certain areas that could delay the growth of *P. ostreatus* (Fig. 4C), [22, 48].

The increase of CO_2_ production in the first 75 days of fermentation is related to the biotransformation of materials easier to degrade such as glucose, present in the solution of trace elements and part of the hydrolyzed yeast, cellulose and hemicellulose, in pine bark and paper napkins (Fig. 4D), [14]. The increase in CO_2_ occurred because, after the O_2_ plasma, the pro-oxidant additives act by favouring the oxidation of the LDPE_oxo_, and low concentrations of CO_2_ are also released [18]; an amount which is in addition to those produced by the biodegradation of the LCB.

The results obtained in this work were similar to those reported by Rojas-Higuera et al. [31], who used a fungal consortium (*Ganoderma lucidum*, *Pleurotus ostreatus*, *Trametes versicolor* and *Phanerochaete chrysosporium*) to biotransform sawdust from *Tabebuia roseae* and *Eucalyptus pellita* at microcosm scale, observing that highest CO_2_ production occurred during the first 45 days; then observed a decrease [31]. Likewise, these results are similar to those of Moreno-Bayona et al. [14], who used the same mixture of LCB used in the present work and observed the production of CO_2_ in two phases. The first phase was lower and lasted until 45 days, the second phase with higher production finished at 75 days [14]. In this work, after day 75, a decrease in the production of CO_2_ was observed in both HMS and VMS because, possibly, lignin and LDPE_oxo_ began to biodegrade since, as they are difficult to degrade compound, *P. ostreatus* takes longer to transform them and therefore the speed of CO_2_ production also decreases. In HMS, the release of CO_2_ behaved with some oscillations, indicating that direct and indirect humification processes were taking place, because of the metabolic activity of *P. ostreatus* and that the different substrates are being metabolized (Fig. 4D).

Ligninolytic enzymes increased as a function of time in both HMS and VMS. However, the trend and activities were different for peroxidases and laccase (Fig. 4E and F). In the HMS, was observed highest peroxidase and Lac activity than in the VMS. Enzyme activity attributed to two aspects, first the distribution of the LCB within the closed system. The thin layer formed by the material into the HMS allowed that fungal colonisation and vegetative growth were higher than in the VMS and promoted the increase of viable and metabolically active fungal biomass; concomitantly with high enzymes activities. In addition, the LCB, being horizontally distributed and thinner than in the VMS, allowed easier diffusion of *P. ostreatus* mycelium and greater access to phenolic and non-phenolic intermediates susceptible to oxidation by LiP in the absence of mediators, especially in the last two samples where the highest activity was observed [54].

On the other hand, polymers such as cellulose and hemicellulose (present in the LCB) were also hydrolysed easier in the HMS system. This situation favoured the formation of low molecular weight intermediates, which are susceptible to be oxidised by auxiliary enzymes involved in lignocellulose degradation. Among these enzymes are glyoxal oxidase (GLOX; EC 1.2.3.5), aryl alcohol oxidases (AAO; EC 1.1.3.7), pyranose 2-oxidase (POX; EC 1.1.3.10), cellobiose dehydrogenase (CDH; EC 1.1.99.18) and glucose oxidase (EC 1.1.3.4). These enzymes produce hydrogen peroxide, released in greater quantity during the last days of processing, allowing it to continue participating as a cofactor for LiP and maintaining the catalytic cycle of the enzyme more efficiently in HMS than in VMS [55].

Authors such as Bellettini et al. [22], reported that during the vegetative growth of *P. ostreatus* these are the enzymes most closely related to the initial colonisation of substrates during the first days of SSF [22]. The second aspect is the availability of O_2_ within the system. Possibly in the HMS, there was a higher concentration of O_2_, which allowed the Lac to have a big number of final electron acceptors, to the formation of water [22, 50]. Another fact favouring Lac activity was the presence of the ABTS redox mediator, which potentiates the enzyme activity and could generate more reactive species, contributing to the oxidation of the LCB. Additionally, cellulases and hemicellulases also participated in the biotransformation process of the LCB. These enzymes release simpler monomers that can be used during primary metabolism and generate intermediates that can promote biological Fenton processes or help regenerate the H_2_O_2_ required during the peroxidase catalytic cycle [27].

The biotransformation of the LCB in both HMS and VMS by *P. ostreatus* and its enzymes were supported by the E4/E6. This ratio makes it possible to semi-quantitatively determine whether total HS formed from an initial organic matter were biotransformed by biotic and abiotic factors related to indirect humification processes [14]. Literature reports that the E4/E6 is inversely proportional to the degree of condensation of the aromatic rings. High values in the E4/E6 suggest a low degree of condensation and the presence of a high proportion of aliphatic compounds. Therefore, by obtaining high values, it is inferred that indirect humification has started and that so far of the FS that is forming in a lower proportion may be recently polymerised SH. As the material matures, the E4/E6 stabilises and may fall below 2.0 when SH predominate [43].

Concerning the present work, results indicate the FS and HS in both microcosm systems after 135 days (Table 1). However, it is not adequate to believe that the LCB was transformed completely and stabilized so that the HS are predominant since the E4/E6 in the HS was not lower than 2.0 (3.07 ± 0.35 and 3.66 ± 0.92 for HMS and VMS, respectively). On the other hand, the finding of strong Ultraviolet spectra (UV) absorption could be due to compounds containing similar chromophore groups (phenolic sands, benzoic acids and anilines derived from polyenes, etc) [56], (Supplementary Material S2).

### Biochar production and characterization

The biochar produced was classified as class III, which means that, under the experimental conditions employed, the percentage of TOC was less than 30%, the FC was 6.55 ± 0.12%, and the ash was 20.2 ± 1.9%. These results indicated that, during the thermal treatment, a high percentage of carbon and other elements were lost in the volatile fraction, decreasing the carbon fraction that should condense as part of the biochar. Additionally, the RM had other mineral-type substances concentrated in the ashes and increased the pH (6.1 ± 0.1) %. The changes produced by the thermal treatment have reported by other authors [14].

### Adsorption studies

The biochar was the product of the co-pyrolysis of a mixture of raw materials, composed of pine bark, napkins, hydrolyzed brewer’s yeast and fungal biomass. Being a heterogeneous mixture, the biochar obtained could have different functional groups that favoured the adsorption of the MG dye at pH 4.0 and 7.0 ± 0.2. The expectation was that MG could be adsorbed by biochar in greater quantity at pH 9.0 ± 0.2 because, at this pH, the biochar surface becomes negative. However, the highest adsorption was observed at pH 4.0 and 7.0 ± 0.2 (0.321 ± 0.003, 0.311 ± 0.023 mg g^− 1^ for each pH), (Table 2). This highest adsorption could be related to the presence of carboxyl groups with a neutral charge and negative charge, which could come from the fungal biomass. These opposite charge groups to the MG dye could participate in the MG adsorption at acid and neutral pH [45, 46]. On the other hand, the biochar ash content could also contribute to the increase of the MG adsorption at pH 4.0 ± 0.2. When biochar containing fungal biomass in a mixture with other materials is produced, the products contain high ash concentration that favour the removal of metals and dyes in solution. Additionally, in biochar produced with pine by-products, the initial pH is close to or higher than the isoelectric point of the biochar, the hydroxyl (−OH) and carboxyl (−COOH) groups of the surface lose protons and becomes negatively charged [57, 58]. Although at the present study, the isoelectric point of the biochar was not determined, possibly the values could be slightly higher than 7.0 ± 0.2. These results, and the pseudo-second-order model, indicate that the adsorption process could occur by chemical attraction (Fig. 5C, Table 2).

Finally, another possible application of the biochar is as a substrate for the germination of seeds of ornamental plants and grass. Table 3 shows that the biochar (T4) without additives serves as a germination substrate and could replace an expensive substrate such as peat. However, the germination percentages can be increased when using PGPB and/or chemical fertilizers. The combined use and low doses of the two products in this study favour the germination process because the bacteria produce substances that promote the germination and development of seeds and seedlings. Afterwards, the germinated seeds can take the nutrients provided by the chemical fertilizer and continue their development due to the porous organic support (biochar), which helps for nutrients retaining [14].

## Conclusions

In this work, the co-biodegradation of LDPE_oxo_ pre-treated with O_2_ plasma and LCB was assumed, by employing *P. ostreatus*, under controlled conditions, in two microcosm systems. In the LDPE_oxo_ sheets post-treated with O_2_ plasma and after 135 days with *P. ostreatus* in the microcosms, the SCA did not return to their initial value concerning a synergistic process between photodegradation and biodegradation caused by the enzymatic activity of the fungus. The geometry of the microcosm influenced the biodegradation of BLC more than that of plastic. The increase of CO_2_ production in the first 75 days of fermentation was related to the biotransformation of materials easier to degrade, such as glucose, present in the trace element solution and then in the LCB. The subsequent increase in CO_2_ was related to the action of pro-oxidant additives present in the LDPE_oxo_, post-treated with O_2_ plasma. In the HMS system, the higher humidity (71.7 ± 1.5%) found at 135 days favoured the growth of *P. ostreatus*, due to the thin layer formed by the content. The thin layer in the HMS, generated better LCB distribution and increase in the contact surface, oxygen transfer, elimination of gases produced by metabolism, as well as higher activity of peroxidase and laccase, compared to the VMS. On the other hand, the biotransformation of LCB increased CO_2_ production and decreased the TOC percentage in the HMS, being this system promising, since it went from an initial TOC of 60.2 ± 2.1% to a final TOC of 14.2 ± 0.2%; while in the VMS it went from an initial TOC of 60.7 ± 1.7% to a final TOC of 20.1 ± 1.3%. Finally, the biochar, produced by the SSF and later pyrolysis, had different functional groups that favoured the adsorption of MG dye at pH 4.0 and 7.0 ± 0.2, besides serving as a substrate for the germination of ornamental plants and grass seeds.

## Supplementary Information

**Additional file 1: Fig. SM1.** UV-VIS spectra of HMS and VMS. **(A)** Abiotic control. **(B)** HMS at initial experiment. **(C)** HMS at 135 d. **(D)** Abiotic control. **(E)** VMs at initial experiment. **(F)** VMS at 135 d. Black line: TMS. Red Line: FS. Blue line: HS.

## Data Availability

All data are available on request.

## Abbreviations

AFM: Atomic Force Microcopy
ANOVA: Analysis of variance
BC: Biochar
BC300: Biochar at 300 °C
CFU: Colony Forming Unit
E_4_/E_6_: Condensation/polymerization ratio
FC: Fixed Carbon
FS: Fulvic substance
FTIR: Fourier Transform Infrared Spectroscopy
GI: Germination Index
HMS: Horizontal Microcosm systems
HS: Humic Substance
*k*: Pseudo-first-order adsorption coefficient
*k*^*2*^: Pseudo-second-order adsorption coefficient
Lac: Laccase
LCB: Lignocellulosic biomass
LDPE: Low-density polyethylene
LDPE_oxo_: Oxo-degradable low-density polyethylene
LiP: Lignin Peroxidase
LTP: Low-Temperature Plasma
MG: Malachite Green
MnP: Manganese Peroxidase
PE: Polyethylene
PGPB: Plant Growth-promoting bacteria
PPA: Personal Protective Accessories
*q*: Amount of dye adsorbed in mg g^− 1^ of biochar
*qe*: maximum amount of dye in mg adsorbed per gram of biochar
R-LCB: Residual Lignocellulosic Biomass
RM: Raw Material
SCA: Static contact angle
SEM: Scanning Electron Microscopy
SSF: Solid State Fermentation
TOC: Total Organic Carbon
UV-VIS: Ultraviolet-Visible spectra
UV: Ultraviolet spectra
V: Visible spectra
VC: Volatile Carbon
VMS: Vertical Microcosm Systems
*α*: Initial adsorption coefficient
*β*: Desorption coefficient

## Declaration

### Concent for publication

All authors agree with the publication of this research.
