# Supplementary material for: Evaluation of two microcosm systems for co-treatment of LDPEoxo and lignocellulosic biomass for biochar production
Source: Biomater Res. 2021 Jul 2;25:21. doi: 10.1186/s40824-021-00222-w (PMC8253244; doi:10.1186/s40824-021-00222-w)
Supplement: Supplementary file 1 — Additional file 1: Fig. SM1. UV-VIS spectra of HMS and VMS. (A) Abiotic control. (B) HMS at initial experiment. (C) HMS at 135 d. (D) Abiotic control. (E) VMs at initial experiment. (F) VMS at 135 d. Black line: TMS. Red Line: FS. Blue line: HS. [file 40824_2021_222_MOESM1_ESM.docx]

**Supplementary Material**

**Evaluation of two microcosm systems for co-treatment of LDPE_oxo_ and lignocellulosic biomass for Biochar production**

**Supplementary Material 1**

**Methodology for the extraction of HS**

The determination of the total HS (THS) was made by taking 4.0 ± 0.1 g of the R-LCB and depositing them in a 50 mL Falcon tube, then 50 mL of 0.5 M sodium hydroxide was added and left in constant agitation for 24 hours at 200 rpm in an orbital Scilogex ® Shaker SK-O330. The supernatant was recovered by filtration using Whatman No 3 paper and a UV-VIS spectral curve was made between 200 and 700 nm, using a quartz cell and distilled water as a target. From the absorbance readings at 465 nm and 665 nm, the E4/E6, associated with HRT, was determined. The analyses were performed in a Thermo Spectronic spectrophotometer Genesys™ 10 V [1-4].

For the determination of FS, 5 mL of the alkaline extraction filtrate (Total HS) was mixed with 5 mL of 6M HCL and left to agitate for 24 hours at 19 ± 2 ºC and 120 r.p.m., using an orbital Scilogex® Shaker SK-O330 at 19 °C. Subsequently, the sample was centrifuged for 10 minutes at 9790 g for 10 minutes and the supernatant was recovered to perform the spectral curves and determine the E_4_/E_6_ condensation ratios in the FS phase. On the other hand, HS were determined in the sediment of the acid extraction by adding 25 mL of NaOH 0.1 M and 25 mL KCl 0.3 M, the mixture was left in constant agitation for 24 hours at 19±2 ºC and 120 r.p.m in an orbital Scilogex ® Shaker SK-O330. The supernatant was recovered by centrifugation for 10 minutes at 9790 g and the same procedure was performed as for the THS and FS [4].

**Supplementary Material 2**

**Spectral curves**

In the abiotic control for HMS and VMS, the highest absorption was observed in the ultraviolet region and there was a decrease in the visible region for THS, FS and HS, respectively. The spectrum of the THS presented the highest intensity of the three in the ultraviolet region and with several peaks that oscillated between 210 and 290 nm (Figs. SM1A, D). This absorption corresponds to the presence of lignin and phenylpropane subunits which are composed of aromatic rings that present greater absorption in the ultraviolet spectrum.

In Figs. SM1B, C, the spectral curves for the initial HMS inoculated with *P. ostreatus* and at 135 days are presented. By adding the pelleted biomass and mixing it with the LCB, there was a decrease in the HHS signal with respect to abiotic control. The FS signal increased in the UV region and the HS were similar to the abiotic control. After 135 days of biotransformation, the THS and FS were slightly increased, with respect to the initial absorption spectra (time zero and inoculated with *P. ostreatus*). HS decreased slightly when compared to the initial spectra. The spectra for HRT were similar to those obtained for HMS. For FS, greater intensity was observed at the beginning and 135 days than in HMS. The HS showed a similar trend and lower intensity at the beginning and 135 days (Figs. SM1E, F).

For the HS spectra in HMS and VMS at 135 days, a discrete shoulder was observed around 220 and 270 nm, which could be associated with the overlap of absorptions of a large number of chromophore groups present in the HS core (Figs. SM1C, F).


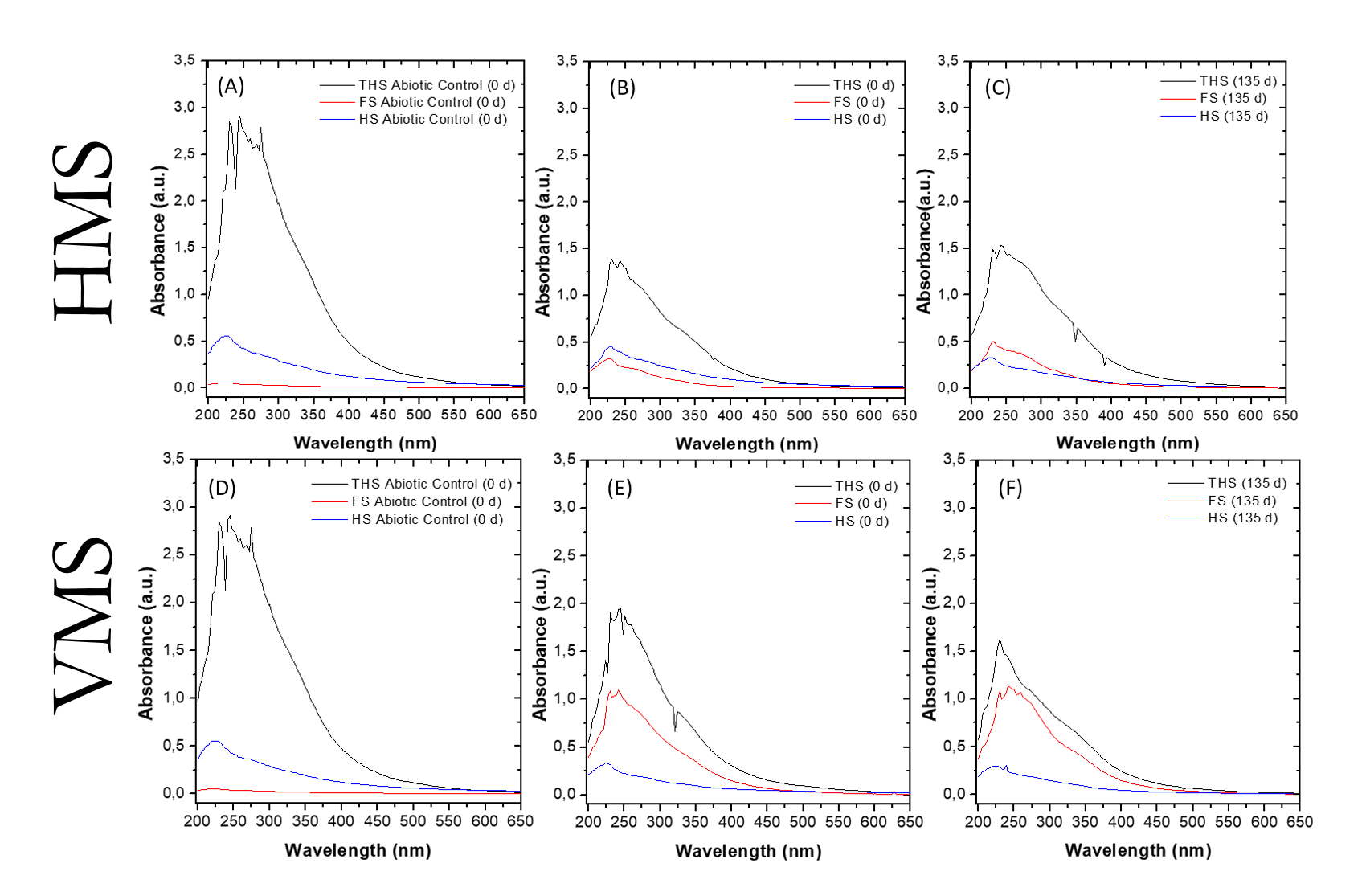


**Fig. SM1** UV-VIS spectra of HMS and VMS. **(A)** Abiotic control. **(B)** HMS at initial experiment. **(C)** HMS at 135 d. **(D)** Abiotic control. **(E)** VMs at initial experiment. **(F)** VMS at 135 d. Black line: TMS. Red Line: FS. Blue line: HS

**References**

1. Gondar D, Lopez R, Fioli S, Antelo JM, Arce F: Characterization and acid-base properties of fulvic and húmic acids isolated from two horizons of an ombrotrophic peat bog. *Geoderma* 2005, 126(3-4):367-374.

2. Helal AA, Murad GA, Helal AA: Characterization of different humic materials by various analytical techniques. *Arab J Chem* 2011, 4:51-54.

3. Moreno-Bayona DA, Gómez-Méndez LD, Blanco-Vargas A, Castillo-Toro A, Herrera-Carlosama L, Poutou-Piñales RA, Salcedo-Reyes JC, Díaz-Ariza LA, Castillo-Carvajal LC, Rojas-Higuera NS *et al*: Simultaneous bioconversion of lignocellulosic residues and oxodegradable polyethylene by *Pleurotus ostreatus* for biochar production, enriched with phosphate solubilizing bacteria for agricultural use. *Plos One* 2019, 14(5):e0217100.

4. González-Osorio H, Sadeghian-Khalajabadi S, Zapata-Hernández RD, Mejía-Muñoz B: Fraccionamiento de la materia orgánica en suelos de la zona cafetera de Caldas. 2008, 59(4):310-320.
